# Irreversible Electroporation of Malignant Hepatic Tumors - Alterations in Venous Structures at Subacute Follow-Up and Evolution at Mid-Term Follow-Up

**DOI:** 10.1371/journal.pone.0135773

**Published:** 2015-08-13

**Authors:** Marco Dollinger, René Müller-Wille, Florian Zeman, Michael Haimerl, Christoph Niessen, Lukas P. Beyer, Sven A. Lang, Andreas Teufel, Christian Stroszczynski, Philipp Wiggermann

**Affiliations:** 1 Department of Radiology, University Medical Center Regensburg, Regensburg, Germany; 2 Center for Clinical Studies, University Medical Center Regensburg, Regensburg, Germany; 3 Department of Surgery, University Medical Center Regensburg, Regensburg, Germany; 4 Department of Internal Medicine I, University Medical Center Regensburg, Regensburg, Germany; University of California at Berkeley, UNITED STATES

## Abstract

**Purpose:**

To evaluate risk factors associated with alterations in venous structures adjacent to an ablation zone after percutaneous irreversible electroporation (IRE) of hepatic malignancies at subacute follow-up (1 to 3 days after IRE) and to describe evolution of these alterations at mid-term follow-up.

**Materials and Methods:**

43 patients (men/women, 32/11; mean age, 60.3 years) were identified in whom venous structures were located within a perimeter of 1.0 cm of the ablation zone at subacute follow-up after IRE of 84 hepatic lesions (primary/secondary hepatic tumors, 31/53). These vessels were retrospectively evaluated by means of pre-interventional and post-interventional contrast-enhanced magnetic resonance imaging or computed tomography or both. Any vascular changes in flow, patency, and diameter were documented. Correlations between vascular change (yes/no) and characteristics of patients, lesions, and ablation procedures were assessed by generalized linear models.

**Results:**

191 venous structures were located within a perimeter of 1.0 cm of the ablation zone: 55 (29%) were encased by the ablation zone, 78 (41%) abutted the ablation zone, and 58 (30%) were located between 0.1 and 1.0 cm from the border of the ablation zone. At subacute follow-up, vascular changes were found in 19 of the 191 vessels (9.9%), with partial portal vein thrombosis in 2, complete portal vein thrombosis in 3, and lumen narrowing in 14 of 19. At follow-up of patients with subacute vessel alterations (mean, 5.7 months; range, 0 to 14 months) thrombosis had resolved in 2 of 5 cases; vessel narrowing had completely resolved in 8 of 14 cases, and partly resolved in 1 of 14 cases. The encasement of a vessel by ablation zone (OR = 6.36, p<0.001), ablation zone being adjacent to a portal vein (OR = 8.94, p<0.001), and the usage of more than 3 IRE probes (OR = 3.60, p = 0.035) were independently associated with post-IRE vessel alterations.

**Conclusion:**

Venous structures located in close proximity to an IRE ablation zone remain largely unaffected by this procedure, and thrombosis is rare.

## Introduction

Thermal ablation of tumors adjacent to major liver vessels remains challenging: on the one hand, because the “heat sink” effect leads to the loss of thermal energy by convection and thus increases the risk of incomplete ablation [1–3], and, on the other hand, because of the risk of vessel damage, especially of the endothelium [4].

Irreversible electroporation (IRE) as a non-thermal ablative option seems to overcome those above mentioned limitations of thermal ablation procedures: histopathological animal studies have shown that IRE induces cell death up to a vessel wall without any perivascular sparing [5–7] whilst preserving the normal architecture of blood vessels [5, 6, 8, 9]. However, mild histopathological changes in adjacent vessel walls as vasculitis and mild endothelial damage have been detected in animal studies [5, 8, 10]. Additionally, alterations of vessels adjacent to an IRE ablation zone were detected by means of computed tomography (CT) imaging after IRE on porcine livers [6]. Similar findings have been reported after IRE ablation of 129 tumors in different organs in humans [11].

The objective of this retrospective study was to evaluate risk factors associated with alterations in venous structures adjacent to an ablation zone after IRE of hepatic malignancies in humans at subacute follow-up. Additionally, the evolution of these changes was evaluated by means of further follow-up imaging.

## Material and Methods

### Study design, participant selection, and patient characteristics

A single-center retrospective observational study was conducted to describe alterations in venous structures after percutaneous IRE of malignant liver tumors evaluated by means of contrast-enhanced magnetic resonance imaging (MRI) or CT. The study was approved by the local ethics committee (Ethics Committee of the University of Regensburg). Each patient signed an informed consent form for the ablation procedure, the acquisition of contrast-enhanced CT or MRI images, or both, and the anonymous use of data for scientific purposes.

Patients were included in this study if they fulfilled the following criteria: (1) Patients were suffering from primary or secondary liver malignancy. (2) Liver tumors were treated by percutaneous IRE. (3) Patients had undergone a contrast-enhanced MRI or CT scan of the whole liver during the portal venous phase before and after each intervention. (4) Post-ablative imaging had to be conducted at the subacute follow-up (i.e. 1 to 3 days after the ablation). (5) Venous structures (portal veins [PVs], hepatic veins [HVs], inferior vena cava [IVC], and transjugular intrahepatic portosystemic shunt [TIPS]) were located within a perimeter of 1 cm of the ablation zone at subacute follow-up.

A prior treatment of liver malignancies by systemic chemotherapy, radiotherapy, surgery, or IRE ablation was no exclusion criterion.

43 patients (32 men and 11 women) aged 60.3 years ± 11.9 (range, 22 to 80 years) who had undergone IRE ablation of 84 hepatic lesions in 63 ablation procedures fulfilled the above mentioned inclusion criteria and were included in this study (Table 1). 31 of the 84 lesions (number of patients, 20) were primary liver tumors, and 53 (number of patients, 23) were secondary liver tumors (Table 2). The mean tumor diameter was 2.4 cm ± 1.1 (range, 0.5 to 6.3 cm). 48 sessions involved the ablation of 1 tumor and 15 sessions the ablation of 2 to 4 tumors.

**Table 1:** Baseline patient characteristics.

Characteristic	
Age (y), mean (SD), range	60.3 (11.9), 22–80
Sex, n (%)	
Male	32 (74.4)
Female	11 (25.6)
Patients with cirrhosis, n (%)	16 (37.2)
Child-Pugh class A	5 (11.6)
Child-Pugh class B	10 (23.3)
Child-Pugh class C	1 (2.3)
History of atherothrombosis^#^, n (%)	7 (16.3)
History of DVT, n (%)	2 (4.7)
History of pulmonary embolism, n (%)	0
Arterial hypertension, n (%)	7 (16.3)
Diabetes mellitus, n (%)	14 (32.6)
Anticoagulative medication, n (%)	13 (30.2)

^#^history of ischemic stroke and/or myocardial infarction and/or aortic valve calcification and/or peripheral artery occlusive disease

**Table 2:** Tumor types in 43 patients treated with irreversible electroporation of malignant liver tumors.

Diagnosis	No. Patients	No. treated lesions
Primary liver tumors		
Hepatocellular carcinoma	16	25
Cholangiocellular carcinoma	4	6
Metastases of		
Colorectal tumor	16	39
Mammarian carcinoma	2	6
Neuroendocrinic tumor	1	2
Other^#^	4	6
**Total**	**43**	**84**

^#^testicular tumor, gastrinoma, esophageal cancer, carcinoma of unknown origin

Two radiologists with 3 years and 9 years of experience in abdominal imaging, respectively, examined each subacute follow-up image for possible vascular changes in diameter, patency, and flow in a consensus reading; the evolution of such vessel alterations was evaluated by means of further follow-up imaging (MRI or CT scans, or both).

### Ablation procedure

All ablation procedures were performed percutaneously under CT fluoroscopy guidance using the NanoKnife System (AngioDynamics, Latham, New York). The IRE parameters were as follows: electric field, 1500 V/cm needle distance; pulse length, 90 μs; pulses per cycle, 70. Post-interventionally all patients received 20 mg enoxaparin subcutaneously once a day until full mobilization.

### Statistical analysis

Data are presented as frequency counts and percentages. To take account for multiple vessels per patient, marginal generalized linear models (GLM) were used to identify variables for prediction of vascular changes at subacute follow-up. The mean response was modeled as a logistic regression model. The binary response for individual patients was assumed to be equally correlated, implying an exchangeable correlation structure. Maximum-likelihood odds-ratio estimators (OR) and 95% confidence intervals are presented as effect estimates. Univariate models as well as one multivariable model, including all variables with p<0.1 in the univariate model were calculated. Associations were considered significant for p<0.05. All statistical analyses were performed with SAS 9.4. Variables analyzed were age (<65y vs ≥65y), sex, the presence of hypertension, anticoagulative therapy, diabetes mellitus, liver cirrhosis, atherosclerosis, status post deep vein thrombosis, nature vessel being adjacent to ablation zone (PV vs HV/IVC/TIPS), and localization of vessel with regard to ablation zone (encased vs abutting/distant).

## Results

**Fig 1:**
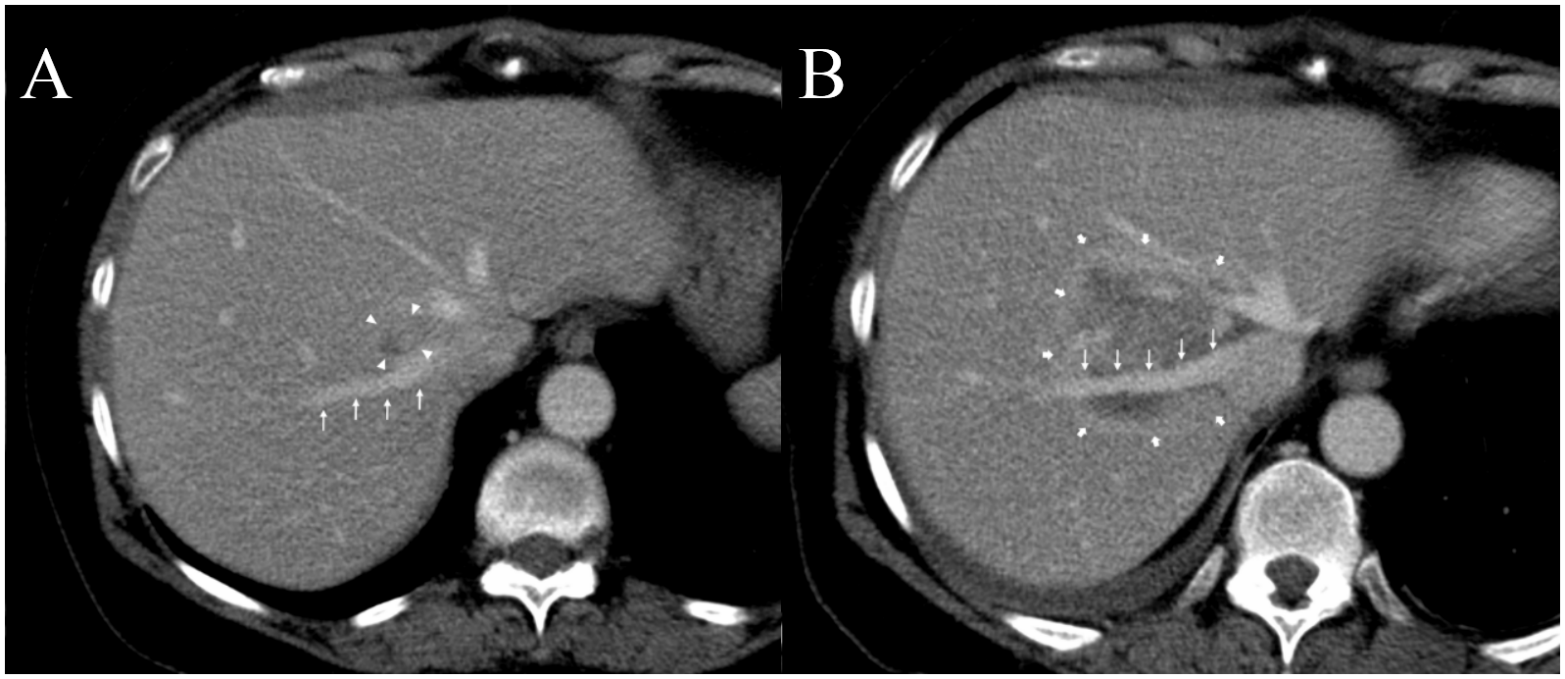
A 71-year old women with centrally located metastasis of colorectal cancer in Couinaud segment VIII of the liver. (A) On pre-interventional contrast-enhanced portal venous image the right hepatic vein (thin arrows) is directly adjacent to the hepatic metastasis (only partly shown on this layer, arrow heads). (B) The post-interventional image 3 days after IRE shows that the right hepatic vein (thin arrows) is encased by the ablation zone (thick arrows), but no vessel narrowing or thrombotic changes are detectable.

**Fig 2:**
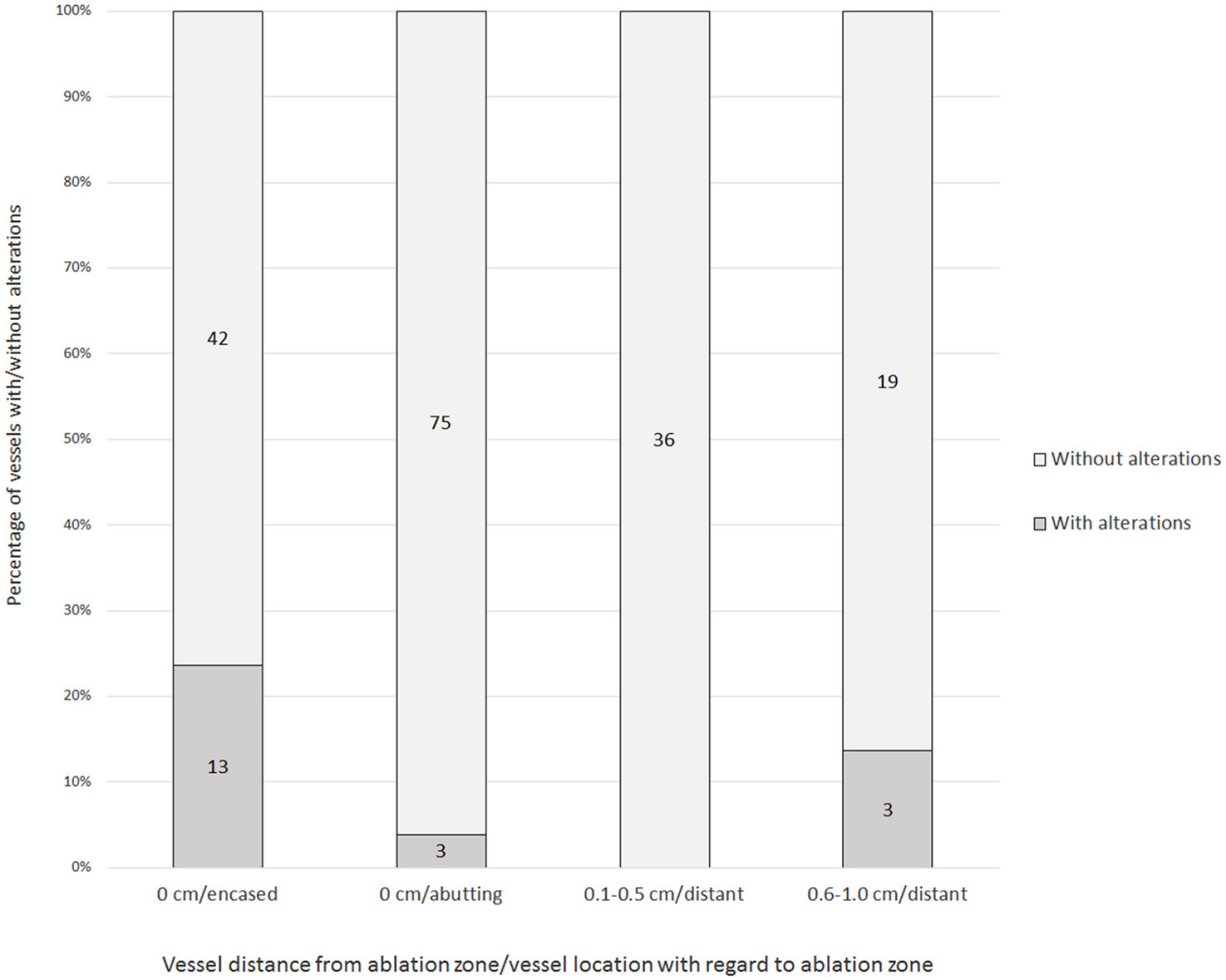
Percentage and absolute numbers of vessels with/without alterations with regard to their location to ablation zone.

**Fig 3:**
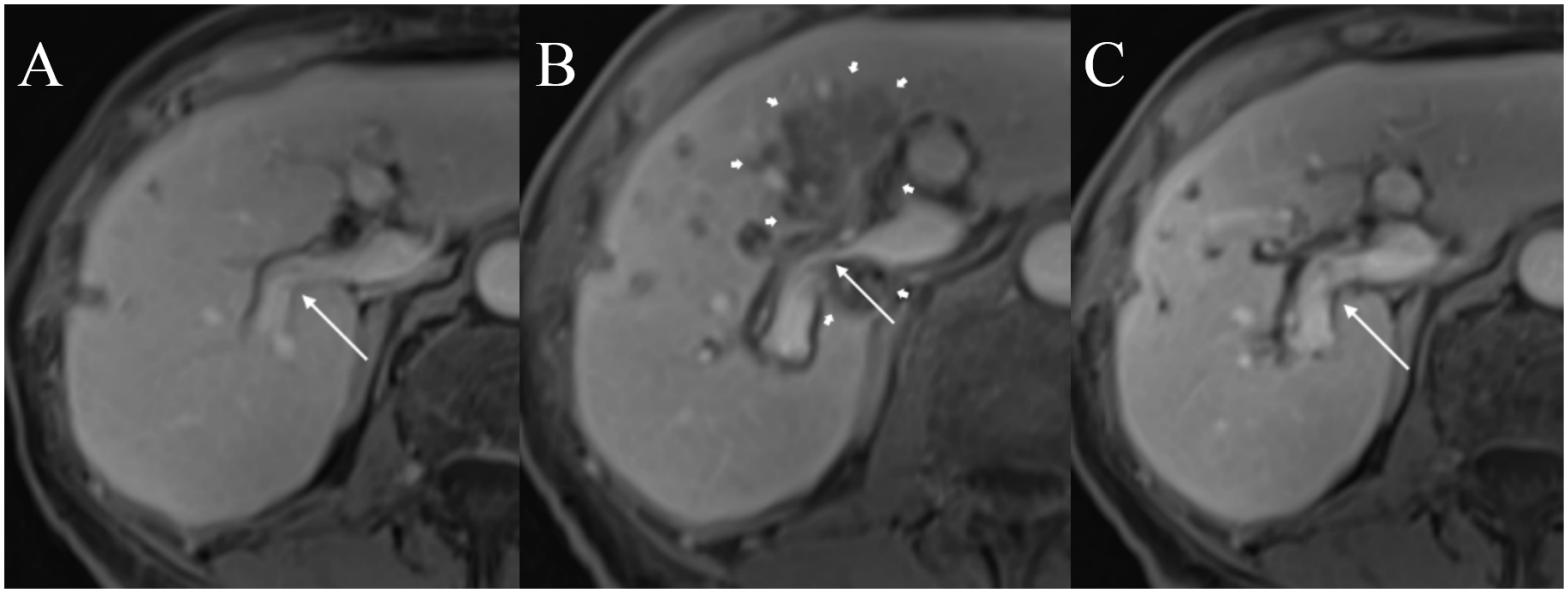
A 63-year old women (patient #4) with centrally located metastasis (not shown) of colorectal cancer. (A) Pre-interventional contrast-enhanced T1w magnetic resonance imaging (MRI) shows a freely perfused right branch of the portal vein (thin arrow). (B) A T1w MRI image obtained on the 3^rd^ post-interventional day shows a caliber reduction of the right portal vein (thin arrow) encased by the ablation zone (thick arrows). (C) 6 weeks after the intervention, the lumen reduction of the right portal vein has resolved (thin arrow).

**Fig 4:**
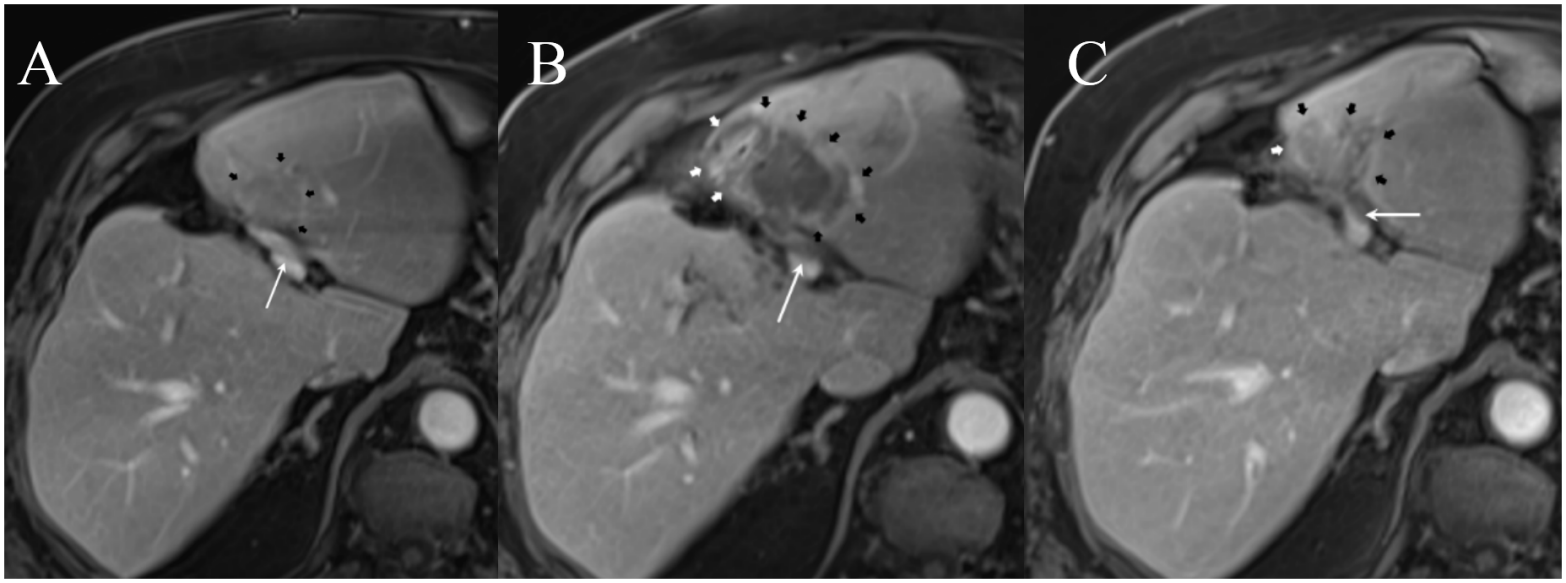
A 75-year old man (patient #15) with hepatocellular carcinoma. (A) Pre-interventional contrast-enhanced T1w magnetic resonance imaging shows a freely perfused portal vein (thin arrow); HCC lesion is partly visible (thick arrows). (B) 1 day after the ablation, the T1w image shows the ablation zone (thick arrows) and newly occurred partial thrombosis of the left portal vein (thin arrow). (C) At the 4-month follow-up, partial thrombosis of the left portal vein has persisted (thin arrow), and the ablation zone has decreased (thick arrows).

In these 43 patients 191 venous structures were located within a perimeter of 1.0 cm of the ablation zone; details regarding vessel location and vessel type are listed in Table 3. At subacute follow-up in 172 of 191 vessels (90.1%) no vascular alteration was noted (Fig 1), in 19 vessels (9.9%) in 17 patients post-IRE alterations were detected (Tables 3 and 4; Fig 2): in particular changes of PV, HV, IVC, and TIPS were noted in 16 of 77 (20.8%), 2 of 96 (2.1%), 1 of 17 (5.9%), and 0 of 1 (0%) of cases, respectively (Table 3). 14 of the 19 alterations (14 of 191 [7.3%]) consisted of vessel narrowing (Fig 3). 2 and 3 of the 19 post-ablative alterations (2 of 191 [1.0%] and 3 of 191 [1.6%)]) consisted of partial and complete PV thrombosis, respectively (Fig 4). The mean period for a further post-interventional follow-up scan after subacute imaging, which was available in 16 of 17 patients, was 5.7 months ± 4.1 (range, 0 to 14 months).

**Table 3:** The type and number of vessels adjacent to the ablation zone and number of vessels with vascular alterations with regard to their localization.

Vessel	Total number of vessels/number of vessels with vascular alterations	Number of vessels in location regarding ablation zone/number of vessels with vascular alterations		
		Encased/altered	Abutting/altered	Within a perimeter of 0.1–1.0 cm/altered
Main PV	7/0	1/0	0/0	6/0
Right PV or segmental PV branch	39/5	13/5	16/0	10/0
Left PV or segmental PV branch	31/11	10/7	9/3	12/1
Middle HV	36/1	13/1	16/0	7/0
Right HV	38/1	13/0	14/0	11/1
Left HV	22/0	5/0	11/0	6/0
IVC	17/1	0/0	11/0	6/1
TIPS stent	1/0	0/0	1/0	0/0
**Total**	**191/19**	**55/13**	**78/3**	**58/3**

PV, portal vein; HV, hepatic vein; IVC, inferior vena cava; TIPS, transjugular intrahepatic portosystemic shunt

**Table 4:** Patients with vascular changes at subacute follow-up imaging (i.e. 1–3 days after ablation), type of altered vessel, location of vessel with regard to IRE ablation zone and IRE needles, kind of vascular alteration, vessel diameter at subacute follow-up imaging and its evolution at follow-up imaging.

Patient No	Vessel	Vessel location with regard to ablation zone^§^	Minimum distance of vessel to IRE needle	Kind of vascular alteration at subacute follow-up	Vessel diameter at subacute follow-up in relation to pre-interventional diameter	Further follow-up	Vessel diameter at further follow-up in relation to pre-interventional diameter	Evolution of subacute vascular alteration at further follow-up
1^#^	rPVsb	e	0.3 cm	Lumen narrowing	57%	3 mo	100%	Resolved
1^#^	mHV	e	0.5 cm	Lumen narrowing	66%	3 mo	100%	Resolved
2*	rPVsb	e	0.6 cm	Lumen narrowing	40%	6 mo	70%	Partly resolved
2*	lPV	d (0.8 cm)	1.5 cm	Partial thrombosis	—	7 mo	—	Resolved
3	rPV	e	0.4 cm	Lumen narrowing	67%	4 mo	100%	Resolved
4	rPV	e	0.2 cm	Lumen narrowing	14%	2 mo	100%	Resolved
5	rPV	e	0.7 cm	Lumen narrowing	66%	11 mo	60%	Progressive lumen narrowing due to local tumor progression
6	lPVsb	a	0.3 cm	Lumen narrowing	50%	3 mo	100%	Resolved
7	lPVsb	e	0.8 cm	Lumen narrowing	33%	6 mo	100%	Resolved
8	lPVsb	e	0.3 cm	Lumen narrowing	66%	9 mo	66%	Persistent lumen narrowing, no local tumor progression
9	lPVsb	e	0.7 cm	Lumen narrowing	66%	0	—	No follow-up due to resection of lPV and left liver lobe as planned before IRE
10	lPV	a	0.5 cm	Complete thrombosis	—	14 mo	—	Thrombosis persisted; volume reduction of left liver lobe; no infarction
11	lPVsb	e	0.6 cm	Complete thrombosis	—	12 mo	—	Infarction of Couinaud liver segments II and III; thrombosis persisted
12	lPVsb	e	0.3 cm	Lumen narrowing	57%	8 mo	100%	Resolved
13	lPVsb	e	0.4 cm	Complete thrombosis	—	7 d	—	Resolved, no infarction
14	lPVsb	e	0.5 cm	Lumen narrowing	63%	4 mo	100%	Resolved
15	lPVsb	a	0.9 cm	Partial thrombosis	—	3 mo	—	Persistent thrombosis; volume reduction of left liver lobe; no infarction
16	rHV	d (1.0 cm)	1.4 cm	Lumen narrowing	56%	2 mo	0	Complete thrombosis secondary to new hepatic mass at the confluence of the rHV and the IVC
17	IVC	d (0.8 cm)	1.0 cm	Lumen narrowing	53%	9 mo	53%	Persistent lumen narrowing, no local tumor progression

IRE, irreversible electroporation; lPV, left portal vein; lPVsb, left portal vein segmental branch; rPV, right portal vein; rPVsb, right portal vein segmental branch; mHV, middle hepatic vein, rHV, right hepatic vein; IVC, inferior vena cava; e, encased; a, abutting; d, within a perimeter of 0.1–1.0 cm; mo, months; d, days;

^#^same procedure,

*different procedures;

^§^in the case of “d” (i.e. within a perimeter of 0.1–1.0 cm) the exact distance from border of the ablation zone is mentioned in parentheses.

In the univariate GLMs, number of IRE needles (≤3 vs. >3), the presence of diabetes mellitus (no vs yes), the type of affected vessel (PV vs HV/IVC/TIPS) and vessel location with regard to ablation zone (encased vs abutting/distant) were significant risk factors for the occurrence of vessel alterations. In the multivariable model number of IRE needles, type of affected vessel and vessel location with regard to ablation zone remained significant (Table 5).

**Table 5:** Results of generalized linear models predicting vessel alterations.

	Univariate models		Multivariable model*	
	Odds Ratio (95%-CI)	p-value	Odds Ratio (95%-CI)	p-value
Age (ref: <65y vs ≥65y)	1.72 (0.84, 3.52)	0.140		
Number of IRE needles (ref: ≤ 3 vs >3)	4.96 (1.13, 21.82)	**0.034**	3.60 (1.09, 11.88)	**0.035**
Sex (ref: male vs female)	1.61 (0.84, 3.08)	0.151		
Hypertension	0.44 (0.07, 2.62)	0.367		
Anticoagulation	1.41 (0.64, 3.13)	0.395		
Diabetes mellitus	2.02 (1.01, 4.05)	**0.048**	1.88 (0.86, 4.08)	0.112
Liver cirrhosis	1.39 (0.66, 2.93)	0.387		
Atherosclerosis	1.98 (0.67, 5.86)	0.215		
S/p DVT	1.71 (0.70, 4.17)	0.240		
Vessel type (ref: no PV vs PV)	10.12 (2.85, 35.97)	**<0.001**	8.94 (2.65, 30.16)	**<0.001**
Vessel location (ref: not encased vs encased)	5.73 (3.02, 10.91)	**<0.001**	6.36 (2.58, 15.65)	**<0.001**

IRE, irreversible electroporation; DVT, deep vein thrombosis; S/p, status post; PV, portal vein; no PV, HV or IVC or TIPS; not encased, abutting or distant;

*includes all variables with p<0.1 in the univariate model.

## Discussion

As a non-thermal ablative method, IRE has shown promising results in ablating perivascular tissue. Preliminary animal studies on the IRE ablation of perivascular porcine liver tissue have shown that this method is not affected by the common heat sink phenomenon [7, 12]. Complete tissue ablation up to the vessel wall could be achieved without any perivascular sparing, and vessels within the ablation zone remained patent at follow-up [6, 10, 12]. Similar results have been reported by Lee and colleagues after IRE ablation of VX2 tumors in rodent livers [13].

A retrospective review of safety and efficacy of IRE in the clinical setting was published by Scheffer and colleagues [14]: Their results included 129 patients in whom hepatic malignancies were ablated by IRE using an open, laparoscopic, or percutaneous approach. In this review portal vein thromboses/occlusions after hepatic IRE had occurred in 1 case in one study: Kingham et al. [15] evaluated safety of IRE ablation of 65 liver tumors being juxtaposed to main hepatic vessels: 25 and 16 tumors were located within a perimeter of 1 cm of a major HV and a major portal pedicle, respectively; only 1 patient had developed post-ablative thrombosis within a portal pedicle. In a recent study not included in the above mentioned review, Narayanan and colleagues [11] have treated 101 patients with 129 lesions, mostly hepatic tumors (100 of 129), with IRE ablation. 158 vessels located within a perimeter of 1 cm of the treatment zone were evaluated in the follow-up; abnormal alterations occurred in 7 of 158 vessels (4.4%) including thrombosis in the portal venous system (n = 4) and mild narrowing of the PV (n = 2) and the HV (n = 1).

The current study focussed on vessel alterations after IRE ablation of hepatic tumors. Post-ablative IRE-related vessel alterations had occurred in 19 of 191 cases (9.9%), mainly consisting of lumen narrowing (14 of 19), which were high-grade stenosis in select cases. After IRE in porcine livers Lee et al. [6] reported lumen narrowing of HVs at subacute follow-up in 39% (9 of 23) of HVs which showed contiguity to IRE lesions. A further follow-up was available in 3 of the 9 cases: 2 of the 3 narrowed HVs had extended to their normal caliber at the 4-week follow-up, but 1 of the 3 narrowed HVs had remained unchanged at the 2-day follow-up. As an underlying reason, Lee and colleagues presumed post-ablative hyperemic, inflammatory, and edematous changes in ablated tissue and the surrounding intact liver parenchyma as reported in preliminary studies [7, 8, 16, 17]. Since moderate caliber reduction of venous structures adjacent to the ablation zone is to be expected because of the above-mentioned changes in ablated tissue and surrounding liver parenchyma at subacute follow-up, the authors of the current study consider such alterations a common occurrence. Post-IRE vessel alterations occurred significantly more frequent if more than 3 IRE electrodes were used (p = 0.0266) and if a vessel was encased by the ablation zone (p < 0.0001) compared to vessels which were distant from or abutting the ablation zone. In the current study 2 of 14 cases of lumen narrowing at subacute follow-up have persisted at 9-month follow-up after ablation, and in a third case lumen narrowing has only partly resolved 6 months after the ablation; in neither of the 3 cases there was local tumor progression. This kind of persistent vessel narrowing could have been caused by scarring after IRE as described in preliminary studies [7, 17].

All thromboses in this study (n = 5) occurred in the portal venous system: 1 of the 5 cases had resolved within 1 week and another case within 6 months. 3 of the 5 cases of thromboses had persisted and resulted in reduced liver volume in each case and in additional partial liver infarction in 1 case. The finding of thromboses within the portal venous system in the current study is in line with the results found by Narayanan et al. [11] who detected post-IRE vascular thrombosis only in PVs (n = 4). The authors proposed a greater vulnerability of PVs to IRE-induced vessel damage due to special flow dynamics within these vessels. The statistical analysis of the current study emphasizes this assumption of Narayanan et al. with PVs being significantly more frequently altered (p = 0.0002) compared to HVs and IVC. These patients with post-IRE portal vein (branch) thrombosis had a mean age of 67.8 y (range, 54–80) with two of them having liver cirrhosis, none of them having a known atherosclerotic disease and two of them were under anticoagulation. Because of the risk of IRE-related thrombosis within the portal venous system, special caution should be exercised in patients with significantly reduced liver volume, for instance after liver resection, and in patients with impaired liver function to avoid liver infarction.

This study has several limitations: One is the difference in imaging modalities during follow-up as well as variations in follow-up periods. Moreover data were analyzed retrospectively and the study group consisted of a heterogeneous patient population with primary and secondary hepatic tumors. Furthermore most patients had undergone a previous tumor treatment with chemotherapy, radiotherapy, or both, which could have damaged blood vessels and consecutively made them more susceptible for further IRE-induced damage.

## Conclusion

The current study showed that venous structures located in close proximity to an IRE ablation zone remain largely unaffected by this procedure and that thrombotic vessel changes are rare.

## Supporting Information

S1 Table Characteristics of patients, ablated tumors and adjacent vessels. (XLSX)
