# Correction: Irreversible Electroporation of Malignant Hepatic Tumors - Alterations in Venous Structures at Subacute Follow-Up and Evolution at Mid-Term Follow-Up

**DOI:** 10.1371/journal.pone.0137480

**Published:** 2015-08-28

**Authors:** Marco Dollinger, René Müller-Wille, Florian Zeman, Michael Haimerl, Christoph Niessen, Lukas P. Beyer, Sven A. Lang, Andreas Teufel, Christian Stroszczynski, Philipp Wiggermann

## Notice of Republication

This article was republished on August 19, 2015, due to the release of confidential or copyrighted material. Please download this article again to view the correct version.
